# Supplementary material for: The non-indigenous dung beetle (Onthophagus nuchicornis) can effectively reproduce using the dung of indigenous eastern North American mammals
Source: PeerJ. 2025 Jan 3;13:e18674. doi: 10.7717/peerj.18674 (PMC11702356; doi:10.7717/peerj.18674)
Supplement: Supplemental Information 6 [file peerj-13-18674-s006.docx]

**Supplementary Table 1: Use and timing of anthelmintic compounds for each species.**

|  | Date of last treatment | Deworming product  (active ingredient(s)) | Individual animals per enclosure^1^ |
| --- | --- | --- | --- |
| Bobcat *(Lynx rufus)* | February 2022 | Panacur (Fenbendazole) | 2 |
| Fox *(Vulpes vulpes)* | February 2022 | Panacur (Fenbendazole) | 2 |
| Moose *(Alces alces)* | February 2022 | Eqvalan Gold (Ivermectin and Praziquantel) | 1 |
| Racoon *(Procyon lotor)* | February 2022 | Panacur (Fenbendazole) | 2 |
| Sheep *(Ovis aries)* | No lifetime deworming | - | 41 |

^1^Individual animals per enclosure denotes the maximum number of individuals for each species contributing to the collected dung.

**Supplementary Table 2: Coefficient of determination values for nutritional parameters and F1 beetle outcomes.** Each value is the r^2^ value for a correlation analysis between the F1 outcome and nutritional parameter variables.

|  | % Dry Matter | % Crude Protein | C:N | % Nitrogen |
| --- | --- | --- | --- | --- |
| Mean # of offspring | 0.15 | 0.17 | 0.17 | 0.13 |
| Mean pronotal width | 0.22 | 0.00 | 0.13 | 0.00 |
| Mean proportion of major males to total males | 0.01 | 0.03 | 0.01 | 0.01 |

**Supplementary Table 3: Quantity and mean depth of brood balls in observation chamber trials.** These trials observed nesting behaviour in *Onthophogus nuchicornis* in 3D printed observation chambers. Each chamber consisted of 2 sheets of plexiglass, approx. 30 cm^2^, held parallel to one another with a small gap in between them, created by a u-shaped plastic frame. The gap between the sheets ranged from 7-10 mm, was filled with sand, and had fresh dung and *O. nuchicornus* beetles added and observed daily.

| Trial | Number of brood balls | Mean depth of brood ball ± SD (cm) |
| --- | --- | --- |
| 1A | 23 |  |
| 1B | 18 |  |
| 2A | 14 |  |
| 2B | 11 |  |
| 2C | 12 |  |
| 3A | 9 |  |
